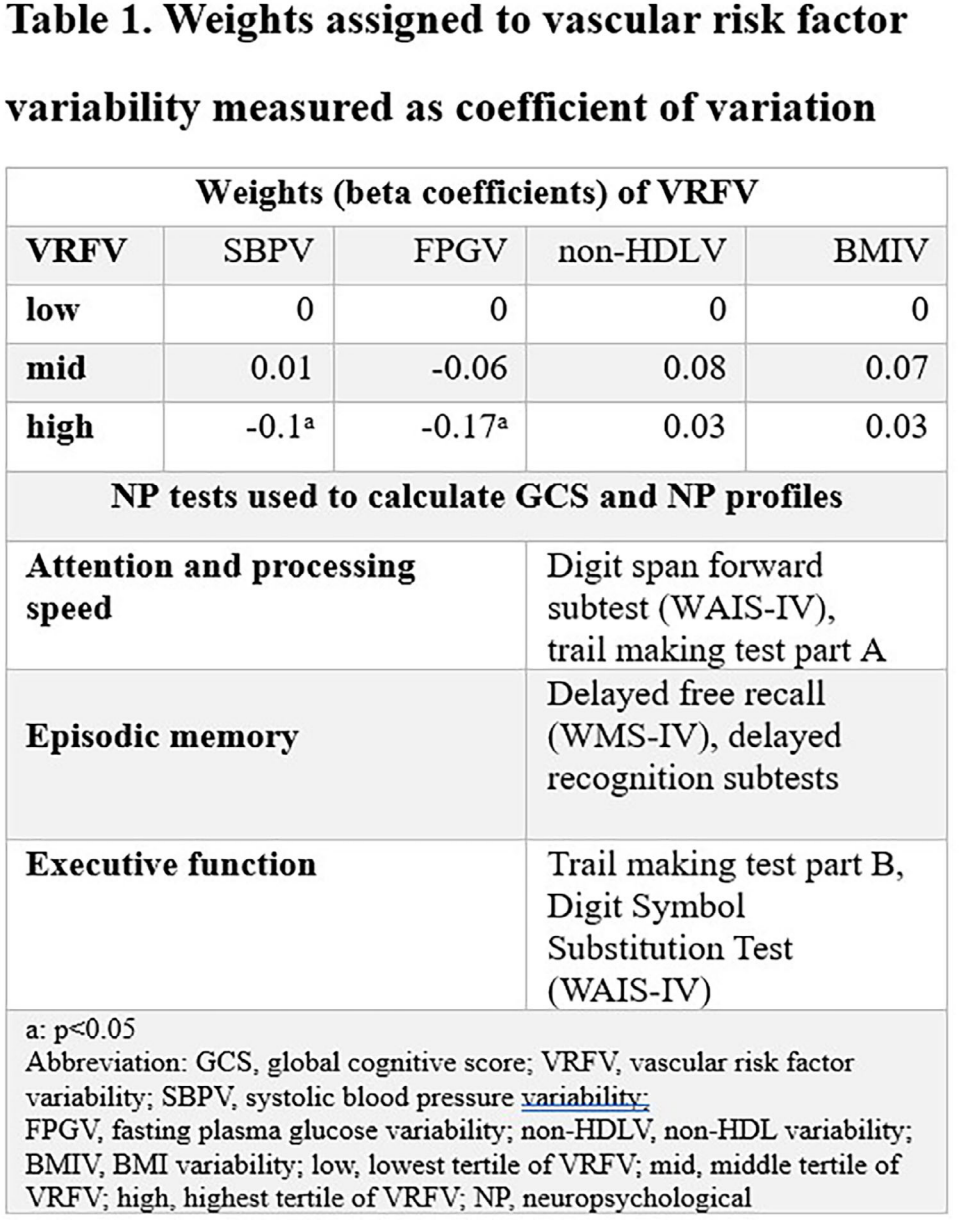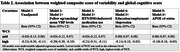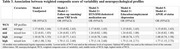# Association of weighted composite score of variability of vascular risk factors with midlife cognitive function: the Bogalusa Heart Study

**DOI:** 10.1002/alz70860_105038

**Published:** 2025-12-23

**Authors:** Soo Jung Kang, Jeanette Gustat, Wan Tang, Phillip H Hwang, Owen T. Carmichael, Ileana De Anda‐Duran, Eunsun Gill, Camilo Fernandez‐Alonso, Lydia A Bazzano

**Affiliations:** ^1^ Celia Scott Weatherhead Tulane University School of Public Health and Tropical Medicine, New Orleans, LA, USA; ^2^ Department of Epidemiology, Boston University School of Public Health, Boston, Boston, MA, USA; ^3^ Pennington Biomedical Research Center, Baton Rouge, LA, USA; ^4^ Tulane University School of Medicine, New Orleans, LA, USA

## Abstract

**Background:**

Variability in levels of individual vascular risk factors (VRFs) from middle age has been associated with increased risk of dementia in later life, independent of mean levels of VRFs. We aimed to investigate the composite and relative impact of variability in multiple VRFs from childhood to adulthood on midlife cognitive function (CF), as this relationship is unclear.

**Method:**

Participants of the Bogalusa Heart Study with ≥ 3 measurements of 4 VRFs from childhood to midlife and assessment of midlife CF were included. Long‐term VRF variability (VRFV) from childhood to midlife was assessed using the coefficient of variation (CV) for systolic blood pressure (SBP), fasting plasma glucose (FPG), non‐high density lipoprotein cholesterol (non‐HDL = total cholesterol ‐ HDL), and body mass index measurements throughout the life course. A weighted composite score of variability (WCS) was created by regressing midlife CF assessed as the global cognitive score (GCS) on tertiles of individual VRFVs (Table 1). The GCS was computed by averaging 8 standardized neuropsychological (NP) test scores. The beta coefficients for each VRFV (weights) were summed to create the WCS. Midlife CF was also assessed as 3 NP profiles by performance on the 8 NP tests (optimal, average, mixed‐low) using cluster analysis. The associations between WCS and both GCS and NP profiles were assessed using multivariable linear regression and multinomial logistic regression models, adjusting for mean VRF levels, smoking, follow‐up, medication use, socioeconomic status, and APOE ε4 status.

**Result:**

Of the 1,010 participants included, 34% were Black, 61% were women, and age at cognitive assessment and follow‐up duration were 48 ± 5 years and 39 ± 3 years. Of the 4 VRFVs, being in the highest tertile of variability in SBP and FPG had the greatest negative influence on midlife CF (Table 1). Compared to the lowest tertile, being in the highest tertile of WCS was associated with a decrease in 0.1 GCS (Table 2) and 77% higher odds of mixed‐low (OR 1.77, 95% CI 1.15, 2.73) compared to optimal midlife CF (Table 3).

**Conclusion:**

Strategies targeting variability of VRFs from childhood to midlife may be beneficial to midlife CF.